# Rapid self-assembly of complex biomolecular architectures during mussel byssus biofabrication

**DOI:** 10.1038/ncomms14539

**Published:** 2017-03-06

**Authors:** Tobias Priemel, Elena Degtyar, Mason N. Dean, Matthew J. Harrington

**Affiliations:** 1 Department of Biomaterials, Max Planck Institute of Colloids and Interfaces, Research Campus Golm, 14424 Potsdam, Germany

## Abstract

Protein-based biogenic materials provide important inspiration for the development of high-performance polymers. The fibrous mussel byssus, for instance, exhibits exceptional wet adhesion, abrasion resistance, toughness and self-healing capacity–properties that arise from an intricate hierarchical organization formed in minutes from a fluid secretion of over 10 different protein precursors. However, a poor understanding of this dynamic biofabrication process has hindered effective translation of byssus design principles into synthetic materials. Here, we explore mussel byssus assembly in *Mytilus edulis* using a synergistic combination of histological staining and confocal Raman microspectroscopy, enabling *in situ* tracking of specific proteins during induced thread formation from soluble precursors to solid fibres. Our findings reveal critical insights into this complex biological manufacturing process, showing that protein precursors spontaneously self-assemble into complex architectures, while maturation proceeds in subsequent regulated steps. Beyond their biological importance, these findings may guide development of advanced materials with biomedical and industrial relevance.

## Abstract (web summary)

Mussels attach to rocks using a byssus, which possesses unique properties of adhesion, toughness and self-healing. Here, the authors explore the fabrication process of mussel byssus demonstrating the self-assembly of specific proteins into multi-scale organized structures using artificially induced byssus threads.

How do living organisms fabricate complex polymeric materials from proteins? In systems from silk to tendon to hagfish slime, answering this complex question has the potential to transform the way humans manufacture polymers. Unlike current polymer processing methods, biological organisms employ bottom-up biomolecular assembly processes to produce a broad range of elaborate nano- and micro-architectured proteinaceous materials, the structural complexity of which is linked to their impressive performance^1^,^2^,^3^. However, there are still enormous gaps in our knowledge of the processing steps involved in the manufacture of complex biomolecular architectures and the relative roles of active and passive assembly processes. This mainly stems from the exceptional technical challenges inherent to observing *in situ* the rapid, localized processes in living biological tissues as they occur. In the present study, we overcome these obstacles by utilizing an innovative experimental approach combining traditional histology and confocal Raman spectroscopic imaging to investigate assembly of the byssus of marine mussels (*Mytilus edulis*).

The mussel byssus is a high-performance fibrous anchor that provides a hedge against dislodgement from crashing waves and hungry predators (Fig. 1)^4^. The byssus represents an ideal model system for investigating biofabrication due to the fact that it is acellular, it assembles and functions outside the body, its formation can be artificially induced to study active assembly processes^5^ and because a great deal is already known about byssus protein composition, structure and function^6^,^7^,^8^. Importantly, development of polymers inspired by the mussel byssus is a fast-emerging field^8^; however, as of yet, these polymers do not match the structural or mechanical complexity of the natural archetype – providing the potential for significant future advances if byssus biofabrication is understood.

A single byssal thread consists of three regions – the core, cuticle and plaque – that are integrated into a cohesive macroscopic fibre, yet possess completely distinct compositions, structural organization and functions (Fig. 1)^6^,^7^,^8^. The byssus core is an energy damping fibrous biopolymer comprised of a semi-crystalline array of collagenous proteins known as preCols (refs 9, 10). The core possesses an initial stiffness comparable to vertebrate tendon (nearly 900 MPa in some species)^11^; however, due to distinctive non-collagenous preCol domains, it is far more tough and extensible, and most notably, exhibits intrinsic self-healing capacity during cyclic loading^12^,^13^. The cuticle, which surrounds the stretchy core, is thought to play a protective role against abrasion due to its unusual combination of high hardness (H=100 MPa for *Mytilus galloprovincialis* similar to an engineering epoxy) and high extensibility (*ɛ*_ult_=70–100%)^14^. The cuticle exhibits a granular morphology resembling that of a particle-reinforced composite^14^,^15^ and is composed largely of mussel foot protein-1 (mfp-1), a repetitive protein rich in a rare post-translational modification of tyrosine called 3,4-dihydroxyphenylalanine (DOPA), which forms metal coordination cross-links with Fe^3+^ ions that contribute more than 80% of the material stiffness and hardness^16^. The plaque, which is also composed of a number of DOPA-rich proteins (mfp-2, -3, -4, -5 and -6) organized into an open-cell foam^17^, is able to adhere under wet conditions to almost any surface chemistry with enough strength to resist the forces produced by crashing waves–a feat unmatched by current industrial glues^8^.

The byssus as a whole functions as an effective anchoring holdfast due to the characteristic material properties of the core, cuticle and plaque, which are dependent on the exact localization, organization and cross-linking of specific protein building blocks into complex hierarchical structures across many length scales^6^,^7^,^8^. Remarkably, each byssal thread is formed in a 3–5 min process, in which more than 10 different types of protein building blocks are secreted and assembled *ex vivo* (Fig. 1)^7^,^18^. Currently, however, the mechanistic understanding of this dynamic assembly process is extremely limited and has advanced little in the last 30 years, aside from recent elegant studies on the formation of the plaque adhesive interface^19^,^20^. The little we do know about this process is principally based on histological and transmission electron microscopy (TEM) investigations, which identified three secretory glands within the mussel foot, each with vesicles of distinctive shape, size and texture^5^,^21^,^22^,^23^,^24^,^25^. During thread assembly, these three glands, which have accumulated an excessive number of names over time, presumably secrete the 11 known protein building blocks of the byssus^26^. However, there has been little direct correlation between the proteins identified in the threads and those present in the glands. At this point, we know only the ingredients and not the full recipe: it remains unclear how the protein building blocks assemble into native thread structure and cross-link within minutes.

Here, we reconcile the decades-old concept of byssus assembly ‘machinery' with the current well-established understanding of byssus structure-function relationships by using traditional histological studies to guide cutting-edge confocal Raman microspectroscopy. Armed with this novel approach, we first localize byssus proteins in the foot tissue glands and then track morphological and chemical changes as they transit from soluble precursors to solid materials during artificially induced thread formation. Our findings reveal a biological workflow of regulated and non-regulated steps: spontaneous self-assembly of complex micro- and nano-scale biomolecular architectures from specific protein building blocks, followed by biologically controlled steps that function during thread maturation (for example, cross-link formation, macroscale molecular alignment). These insights into the byssus manufacturing process clarify the complementary roles and interactions of physically driven versus biologically controlled processing steps in natural material fabrication, with clear potential implications for the production of man-made polymers.

## Results

### Histological staining of foot glands

**Figure 1:**
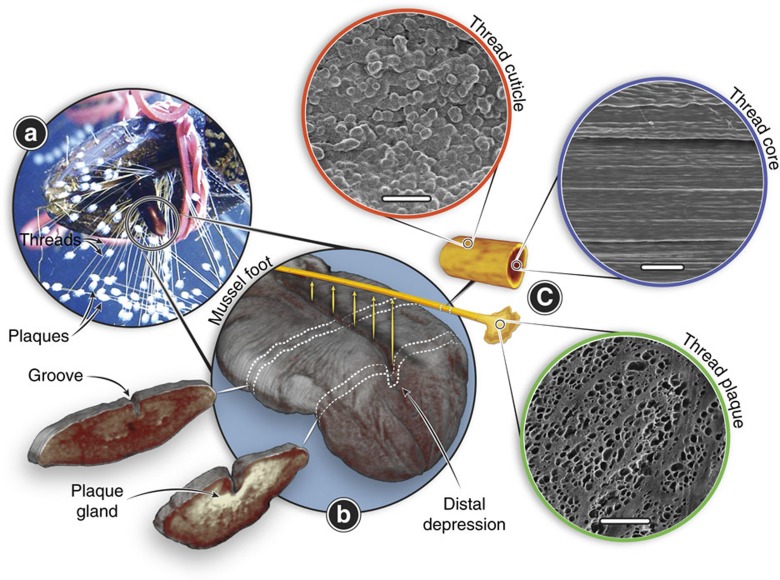
Morphology and fabrication of the mussel byssus. (**a**) Photo of a mussel in the process of extruding threads onto a plexiglass surface with its foot. Note the many threads already deposited. (**b**) μ-CT image of the ventral surface of an iodine-stained mussel foot with two sections cut out to show the inner gland and groove structure in the middle of the foot and near the distal depression. Byssal threads are synthesized one at a time, by secreting protein building blocks into the groove on the ventral side of the mussel foot. (**c**) Schematic of a single byssal thread, with scanning electron microscopy images highlighting the complex micron-scale morphologies of the protective cuticle (scale bar, 3 μm), fibrous core (scale bar, 5 μm) and the adhesive plaque (scale bar, 50 μm).

**Figure 2:**
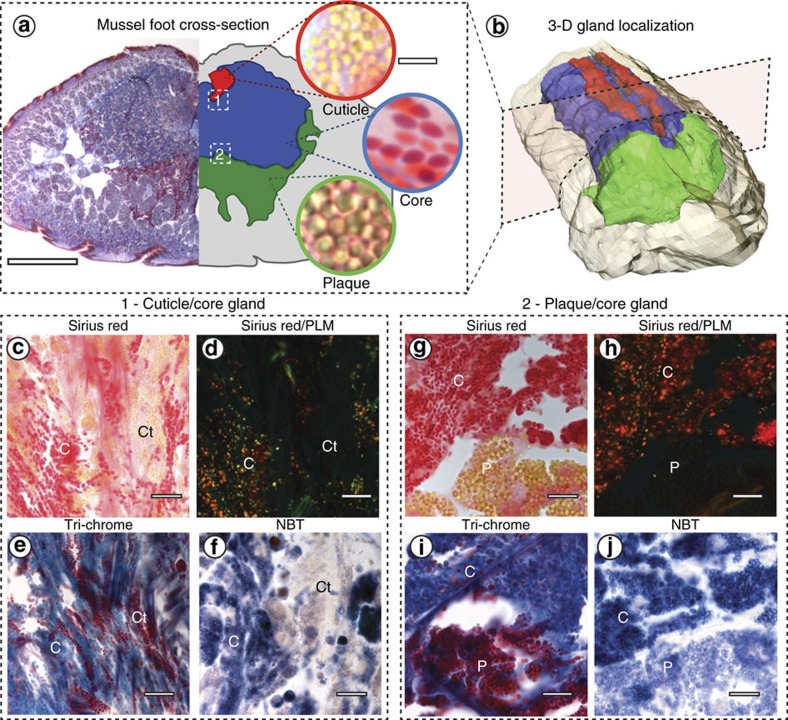
Histological investigation of mussel foot secretory glands. (**a**) Trichrome stained transverse cross-section of the mussel foot tissue (left; scale bar, 1 mm) and accompanying colour-coded illustration showing localization of the three secretory glands (right). High magnification light microscopy images of the vesicles in each gland are shown on the far right (scale bar, 3 μm for all three images). (**b**) 3D reconstruction of a mussel foot in the resting state made from serial trichrome stained transverse foot sections showing gland distribution using the same colour scheme shown in **a**. The colour scheme is arbitrary and is used throughout the paper to identify the specific glands and their contents. (**c**–**j**) Microscopy images of histologically stained mussel foot sections from regions approximately indicated by the numbered white dashed boxes in **a**. Panels **c**–**f** show regions from the interface between the cuticle (Ct) and core (C) glands (box 1), while panels **g**–**j** show regions from interface of the plaque (P) and core (C) glands (box 2). (**c**,**g**) Sirius red stains collagen bright red. (**d**,**h**) PLM imaging of the same region in **c** and **g**, respectively, showing the birefringence of the vesicles in the core gland indicating collagen alignment. (**e**,**i**) Masson's trichrome stains the core gland vesicles blue indicating presence of collagen, whereas the cuticle and plaque vesicles are stained red. (**f**,**j**) Positive, purple-blue coloration with NBT-glycinate staining indicates the presence of oxidized DOPA groups (that is, DOPA-quinone). Scale bars for **c**–**j** are 10 μm.

Byssal threads are produced by a specialized organ called the mussel foot (Figs 1 and 2, Supplementary Movie 1). The tongue-shaped foot from *M. eduli*s is 1–1.5 cm in length in the resting state, but during thread assembly can extend several times its original length (Fig. 1a,b). The ventral groove is a trench-like channel running from the base to the tip along the topside of the foot, where it terminates into a small divot, known as the distal depression (Fig. 1b). The ventral groove provides the mold in which the core and cuticle are formed, whereas the distal depression is the site of plaque formation^18^. Aside from the musculature, the bulk of the foot tissue is comprised of secretory glands surrounding the ventral groove and distal depression, in which the thread proteins are stored in secretory vesicles (Figs 1b and 2).

A key first step in elucidating the dynamic byssus assembly process is the localization of specific byssus proteins in the different foot glands (Table 1). For this purpose, mussel foot cryo-sections were treated with histological stains with affinities for certain byssus proteins (Fig. 2). For example, we utilized two common stains for connective tissues–namely, Masson's trichrome and Sirius red–as preCols, comprising the majority of the thread core by weight, are ∼50% collagen by sequence^10^. Masson's trichrome stains collagen bright blue and most other proteins red^27^, whereas Sirius red stains collagen bright red with high specificity and enhances birefringence of aligned collagen molecules in polarized light microscopy (PLM)^28^. Because most of the cuticle and plaque proteins contain elevated concentrations of DOPA (Table 1), foot sections were also treated with nitro-blue tetrazolium chloride (NBT)-glycinate stain, which turns dark blue in the presence of quinone moieties (for example, oxidized DOPA)^29^ and was previously used to identify DOPA-rich proteins in mussel foot extractions^30^.

Trichrome stained transverse cryo-sections of the distal mussel foot (Fig. 2a) revealed three glands with distinct staining signatures, similar in location to descriptions given in previous studies^5^,^21^,^22^,^23^. We provide evidence, however, that directly links the protein contents of each of the three glands to the core, cuticle or plaque of the thread. As gland names from previous studies^26^ have little correspondence to the functional roles we demonstrate in this study (Table 1), we designate the glands here as the core, cuticle and plaque glands, respectively, and use these names throughout the paper (Fig. 2a, Table 1).

Serial stained cryo-sections made along the entire mussel foot every 200 μm were digitally reconstructed into a high-resolution three-dimensional rendering, where each gland is arbitrarily assigned a specific colour that will be used throughout the paper (Fig. 2b, Supplementary Fig. 1). The plaque gland (shown in green; formerly phenol gland or purple gland) is localized near the distal depression, where the plaque is formed^5^. The core gland (blue; formerly collagen gland or white gland), believed to form the fibrous inner region of the thread^22^,^23^,^24^, is present along the entire length of the foot and comprises the largest volume. The cuticle gland(s) (red; formerly enzyme gland or accessory gland), which was originally thought to contain enzymes necessary for thread sclerotization^23^,^31^, but is now believed to form the cuticle^25^, is interdigitated with the core gland, confined to two thin strips on either side of the ventral foot groove. This three-dimensional (3D) rendering presents a more accurate and detailed depiction than previous hand-drawn illustrations^21^,^22^, but also provides a critical roadmap for the Raman imaging experiments described in the next sections.

Each gland contains specific secretory vesicles with distinctive sizes and morphologies, shown in Fig. 2a. The ovoid core gland vesicles stain bright blue with Masson's trichrome and bright red with Sirius red, indicating the presence of collagenous proteins, likely the preCols^27^,^28^. The underlying cytoplasmic material is identified by its light pink colour, while fibrous collagen in the connective tissue also stains red, but is easily differentiated from the vesicles^10^. Strong birefringence in PLM indicates that the collagenous molecules in the core vesicles are well-aligned (Fig. 2d,h), which is consistent with previous TEM micrographs^5^,^24^ and supports the hypothesis that preCols are stored as a smectic liquid crystalline phase (that is, a fluid phase in which molecules are aligned as books on a shelf)^7^,^32^,^33^.

In contrast to the core vesicles, the spherical cuticle and plaque vesicles contain non-collagenous proteins that are not well-aligned, based on the fact that they stain red with Masson's trichrome, do not stain with Sirius red and do not exhibit birefringence (Fig. 2c–j). Consistent with previous TEM studies^23^,^25^, the spherical vesicles of the plaque gland have a larger diameter (1–2 μm) than the cuticle gland vesicles (0.5–1 μm; Fig. 2a). Core, plaque and cuticle vesicles all stained positive with the NBT-glycinate assay indicating the presence of quinone moieties, likely arising from oxidized DOPA-containing proteins (Fig. 2f,j). Notably, however, the core gland vesicles stained the darkest with NBT, followed by the plaque gland and the cuticle gland. This was unexpected considering that the preCol and TMP-1 proteins thought to be in the core vesicles have a lower DOPA content (1–5 mol%) compared to the proteins comprising the cuticle (15 mol%) and plaque (between 2 and 30 mol%) (Table 1). However, because the intensity of NBT-glycinate staining depends on the amount of the oxidized quinone form of DOPA^29^,^34^ and not just total DOPA content, we posit that the cuticle and plaque vesicles may possess lower levels of DOPA oxidation, perhaps the result of different local conditions. In support, there is strong evidence that in the plaque secretion, the oxidation of DOPA is actively hindered both by the low ambient pH∼2 (ref. 35) and the presence of the thiol-rich anti-oxidant protein mfp-6 (refs 20, 36); however, verification of this hypothesis, also in the other vesicle types, requires further investigation.

### Raman spectroscopy of foot glands

Histology provides important determination of the boundaries of glands and localization of their vesicles in the mussel foot. To obtain specific chemical and structural information about proteins within the glands, we utilized confocal Raman microspectroscopy, a form of vibrational spectroscopy that has been critical for elucidating structure-function relationships in the native byssus^15^,^37^. Here, Raman measurements enabled acquisition of spectral fingerprints from protein molecules in the foot tissue with sub-micron resolution. The combination of spectroscopic and histological maps of the foot enables assignment of specific byssus proteins to the different vesicles – information, which is crucial for studying dynamic assembly processes.

**Figure 3:**
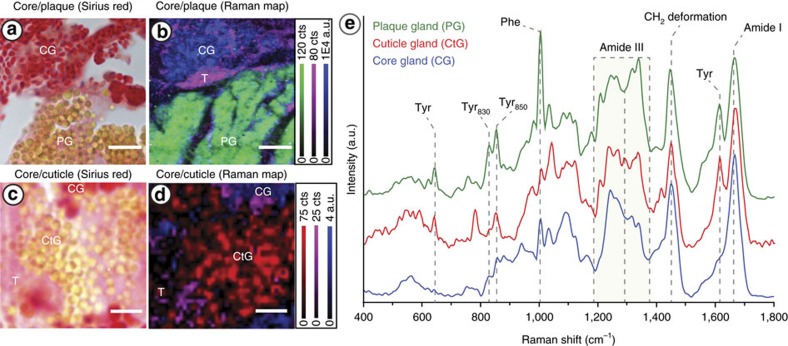
Raman confocal microspectroscopy of mussel foot glands. (**a**) Sirius red staining and (**b**) composite Raman image of an analogous region of the core/plaque gland interface. The composite Raman confocal spectroscopic image was integrated over three spectral regions, which were found to correspond to the core gland (CG) (Blue; ratio of integration of 1,200–1,292 cm^−1^ to 1,292–1,377 cm^−1^), the plaque gland (PG; green; 1,600–1,631 cm^−1^) and surrounding tissue (T; violet; 1,550–1,600 cm^−1^). (**c**) Sirius red staining and (**d**) composite Raman image of an analogous region of the core/cuticle gland interface The composite Raman confocal spectroscopic image was integrated over three spectral regions corresponding to the cuticle gland (CtG; red; 1,600–1,631 cm^−1^), core gland (CG; blue; ratio of integration of 1,200–1,292 cm^−1^ to 1,292–1,377 cm^−1^) and surrounding tissue (T; violet; 1,550–1,600 cm^−1^). Scale bar is 8 μm for panels **a** and **b**, and 3 μm for **c** and **d**. (**e**) Average spectra from each of the secretory glands showing a distinctive spectral fingerprint and thus, different protein composition.

Because Raman measurements must be performed on unstained sections in which it is difficult to identify specific glands, an adjacent serial section from the same mussel foot was first stained with Sirius red to locate regions of interest at the core/plaque gland and core/cuticle gland interfaces (Fig. 3a,c). Raman spectra were acquired from each pixel of analogous regions of interest in the unstained serial section with 0.5 μm resolution. Examination of individual spectra from the Raman maps revealed distinctive spectral fingerprints in different regions, consistent with both collagenous proteins (for example, core proteins) and Tyr-rich proteins (for example, cuticle and plaque proteins). By integrating the intensity of Raman bands specific for collagenous proteins and Tyr-rich proteins in each spectrum, we generated composite two-dimensional maps showing their localization in the image–essentially, creating a spectroscopic histological image (Fig. 3b,d). Notably, spectra of collagen versus Tyr-rich proteins showed analogous distribution to staining patterns for the core gland and the cuticle/plaque glands, respectively, in adjacent Sirius red-stained sections, thus validating the approach. From these maps, averaged Raman spectra of the three different vesicles and underlying tissue were extracted (Fig. 3e), establishing the chemical identity of each gland's vesicles.

By comparing the specific features of the Raman spectra from the different vesicles, we can extract important information about the biochemistry and structure of the contents and, by linking this to the existing knowledge of thread proteins, the destiny of the vesicles in the thread manufacture process. All major bands observed in the Raman spectra from the three vesicle types are consistent with a primarily proteinaceous composition, especially the amide I (1,630–1,700 cm^−1^) and amide III (1,200–1,350 cm^−1^) bands, which provide information about protein backbone conformation^38^,^39^. All three gland spectra indicate extended backbone structure based on their similar amide I band position (∼1,667 cm^−1^); however, a higher degree of conformational order in the proteins of the core gland is indicated by the sharper peak shape^38^. Variations in the relative intensities of the four roughly defined peaks present in the amide III band (∼1,243, 1,269, 1,315 and 1,340 cm^−1^) also indicate protein structural differences. Specifically, core gland proteins exhibit a primarily collagen triple helical or β-sheet conformation consistent with known preCol secondary structures^40^,^41^ based on the higher intensity of the peaks at 1,243 and 1,269 cm^−1^ relative to the other two peaks. In contrast, a less ordered protein conformation in the plaque and cuticle vesicles is indicated by the lower relative intensity of these peaks^39^,^42^,^43^. In light of the histological evidence, the Raman measurements confirm the presence of aligned preCol proteins in the core vesicles and are consistent with the predicted structural disorder of the plaque and cuticle proteins (that is, mfp-1 – mfp-6)^44^.

Aside from being disordered, the proteins that comprise the plaque and cuticle are distinctive because of their elevated composition of the amino acid tyrosine (Tyr), some of which is post-translationally converted to DOPA (Table 1)^8^. Thus, based on the notable Tyr (1,615 cm^−1^, a doublet at 829 and 850 and 643 cm^−1^) and DOPA (785 cm^−1^) Raman peaks observed in the respective spectra, we deduce the presence of DOPA-rich proteins and their precursors in the cuticle (mfp-1) and plaque (mfp-2–6) vesicles. Furthermore, the additional presence of the amino acid phenylalanine (Phe) in plaque vesicles based on the strong peak at 1,006 cm^−1^ specifically indicates the presence of mfp-2, which is the most abundant component of the plaque foam^37^.

Considering that interactions between DOPA residues and metal ions in the plaque and cuticle are critical for byssus function^15^,^37^, the absence in plaque and cuticle vesicle spectra of the distinctive and intense DOPA-metal resonance Raman peaks characteristic of the native plaque and cuticle^15^,^37^ is highly conspicuous, especially considering that both glands stained positive for DOPA with NBT (Fig. 2f,j). Because of the extremely high sensitivity of this Raman resonance signal to even very small amounts of DOPA-metal complexes over a large pH range^45^, we conclude that the metal ions that normally interact with DOPA in native thread cuticle and plaque (for example, Fe and V)^16^ are not stored together with the DOPA-rich proteins in the gland vesicles. In fact, it was previously predicted that cuticle and plaque proteins would not be stored together with metal ions, based on the observation that DOPA residues in purified plaque proteins undergo spontaneous oxidation and covalent cross-linking, when mixed *in vitro* with Fe^3+^ under acidic conditions similar to those in the secretory vesicles^35^,^46^. This has important implications for the way that the plaque and cuticle assemble, which will be discussed later.

### Tracking thread assembly with induced threads

**Figure 4:**
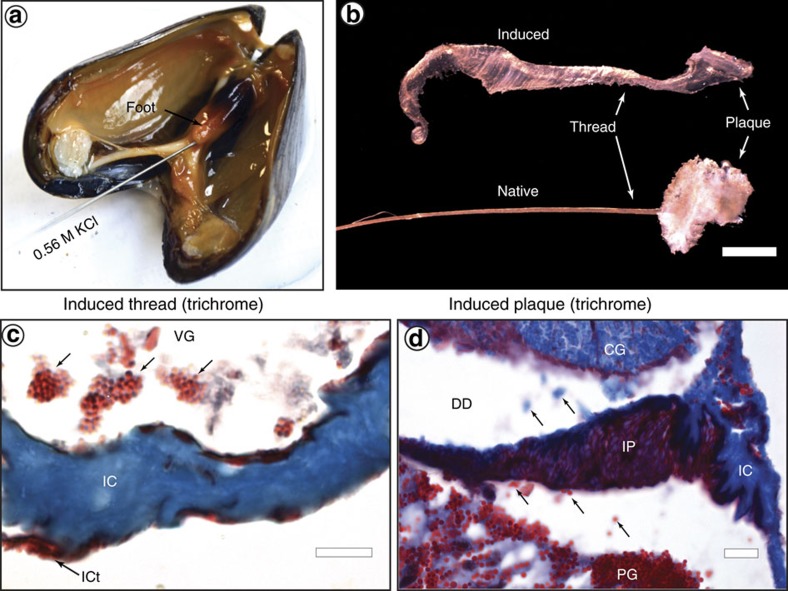
Induced thread formation. (**a**) Injection of KCl into the foot induces secretion of byssal thread secretory vesicles. (**b**) Induced thread morphology is impaired compared to native threads (scale bar, 1 mm). (**c**) Trichrome-stained section of an induced foot within the ventral groove (VG) showing the self-assembly of the induced thread core (IC) and cuticle (ICt). Small arrows indicate vesicles from the cuticle gland in the ventral groove prior to assembly on the core surface (scale bar, 10 μm). (**d**) Trichrome-stained section of an induced foot within the distal depression (DD) showing the self-assembly of the induced thread plaque foam (IP) and core (IC) with cross-sectional morphology similar to that of a native thread. Small arrows indicate secretory vesicles from the core gland (CG) and plaque gland (PG) in the distal depression as they move towards the tissue assembly point (scale bar, 10 μm).

The combined histological and Raman-based investigations of the vesicles bridge older TEM/histological studies with more recent biochemical investigations of byssus proteins; however, they still present a very static perspective of assembly. To obtain a more dynamic view, we employed our combined histological/Raman spectroscopic approach to investigate artificially induced thread formation, which can be achieved by injecting 0.56 M KCl into the pedal nerve at the base of the foot^5^,^47^, leading to muscle contraction and vesicle secretion into the foot groove (Fig. 4a). This procedure was recently exploited to investigate the adhesive secretion in various mussel species^19^,^20^. Figure 4b shows that induced threads are severely morphologically impaired compared to native threads. Considering that the foot is essentially paralyzed during induction, we assume, as previous authors have^17^, that induced thread formation represents spontaneous rather than biologically regulated aspects of the assembly process. By investigating artificially induced thread formation, we are able to observe transport and changes in microstructure of vesicles (via histological staining), as well as chemical and nano-structural changes in byssal thread proteins (via Raman spectroscopy) as vesicle-bound soluble precursors transition to organized tissues. Whereas examination of induced thread formation provides information about unregulated self-assembly of byssal proteins, comparison with native threads highlights biologically regulated steps, allowing dissection of the relative contributions of biological versus physicochemical forces driving byssus manufacture.

By flash freezing dissected mussel feet within 5–10 min after induction with KCl, we were able to capture the thread formation process in progress (in comparison, previous studies collected fully formed induced threads after 30 min^47^). Trichrome-stained cryo-sections from within the ventral groove and from the distal depression of induced feet reveal formation of a nascent induced thread in which core and cuticle (Fig. 4c) and core and plaque (Fig. 4d) assembly are observed. Notably, the individual secretory vesicles in both regions can be seen coalescing to form tissues with microstructured morphologies resembling native thread tissues (Fig. 4c,d), indicating that much of the complexity of byssal thread tissue architecture is formed by unregulated and spontaneous self-assembly. The fact that vesicles coalesce only within the ventral groove or distal depression and not in the glands where they are densely packed supports the hypothesis that the physical conditions (for example, pH, ionic strength) within the groove help initiate assembly^7^,^32^,^33^,^35^. In the following sections, we address assembly of the induced core, cuticle and plaque separately and at a higher resolution, as a means of elucidating the finer scale aspects of composition and structure relevant for byssus fabrication.

### Core assembly

**Figure 5:**
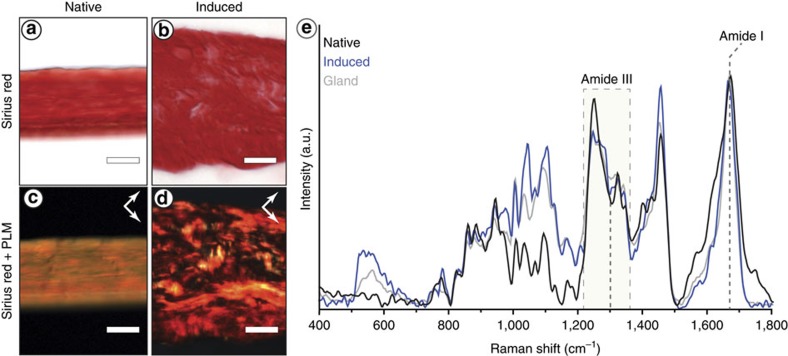
Histological and Raman characterization of induced thread core. (**a**,**b**) Sirius red-stained longitudinal cryo-section of a (**a**) native thread and (**b**) induced thread. (**c**,**d**) PLM images of the same region in (**a**) and (**b**) showing differences in birefringence and thus, protein alignment. Arrows show the orientation of the crossed polarizers. (**e**) Raman spectra from the core gland vesicles, induced core and native core, highlighting the differences in native thread spectra. Scale bar is 20 μm for panels **a**–**d**.

The native byssal thread core consists of a semi-crystalline array of preCol molecules aligned over a length scale of centimeters^9^, leading to strong birefringence in PLM (Fig. 5c). The induced thread core also exhibits birefringence (Fig. 5d); however, it is patchy and restricted to regions of tens of microns, suggesting that local self-assembly of preCols into nano-architectured arrays is spontaneous, likely dictated by the liquid crystalline pre-ordering of molecules in the secretory vesicles^24^,^32^,^33^, whereas longer scale molecular ordering requires biologically regulated processing. Compared to the Raman spectra of the induced thread core, native thread core spectra indicate an increased content of collagen and/or β-sheet conformation based on the higher relative intensity of amide III bands at 1,240 and 1,270 cm^−1^ and the presence of a shoulder at ∼1,630 cm^−1^ in the amide I band^42^,^43^ (Fig. 5e). Furthermore, polarized Raman spectra of induced threads also indicate lower molecular alignment of preCol proteins within the induced threads relative to native threads based on the smaller dependence of laser polarization on the intensity of the amide I band (Supplementary Fig. 2).

These data suggest that induced thread formation lacks certain regulated steps critical for attaining native preCol conformation and alignment. Fibrous biological materials, such as silk and collagen, require careful control of ambient conditions (pH and ionic strength) or application of mechanical forces to attain native structure^48^,^49^. Along these lines, peptides based on terminal preCol cross-linking domains were found to undergo a sudden transition from disordered to highly ordered protein structure when brought from the acidic pH at which the preCols are thought to be stored (pH ∼4) up to the slightly basic pH of seawater (pH ∼8)^50^. This suggests that proper core self-assembly may depend on changes in the ambient environment during protein secretion.

Given that the foot is essentially paralyzed during the thread induction, it is highly feasible that mechanical forces are also necessary to facilitate proper folding and alignment of preCol molecules (for example, from peristaltic foot contractions or mechanical loading) as suggested by Waite^18^. In fact, careful observation of the natural formation process reveals that the mussel foot undergoes distinct muscular contractions during thread formation and immediately prior to releasing the nascent thread from the foot groove (Supplementary Movie 1). The role of mechanical force in aligning preCols during assembly is also supported by studies showing that dilute solutions of purified preCol proteins can be easily hand-drawn into aligned fibres^32^,^51^. Similarly, poly-aramid polymer fibre formation from liquid crystalline solutions also requires mechanical drawing, with the degree of alignment and mechanical performance closely correlated to the draw ratio (that is, the degree to which it is pre-stretched during fabrication)^52^,^53^. In any case, while our findings do support an important role for the liquid crystalline pre-organization of preCol molecules for self-assembly, this alone is clearly not sufficient to create centimetre scale ordered polymer fibres.

### Cuticle assembly

**Figure 6:**
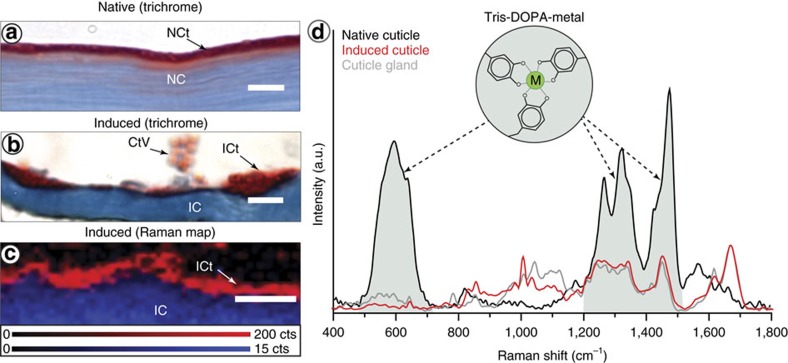
Histological and Raman characterization of induced thread cuticle. (**a**) Trichrome-stained native thread section with blue-staining core (NC) and red-staining granular cuticle (NCt). Scale bar is 4 μm. (**b**) Trichrome-stained section of induced thread core and cuticle with blue-staining core (IC) and red-staining cuticle (ICt). Clustered cuticle vesicles (CtV) can be seen in the ventral groove before assembly. Scale bar is 4 μm. (**c**) Composite Raman confocal spectroscopic image of an induced thread integrated over two spectral regions corresponding to the induced core (blue; 1,226–1,299 cm^−1^) and the induced cuticle (red; 1,600–1,630 cm^−1^). Scale bar is 3 μm. (**d**) Comparison of Raman spectra from native cuticle, induced cuticle and cuticle gland. The intensities of the gland and induced spectra are normalized to the Amide I band height; however, the native cuticle spectra is not normalized due to the extremely high intensity of the DOPA-metal resonance bands.

In native threads, the fibrous core is sheathed by the protective cuticle, which exhibits a knobby structure comprised of hard granules embedded in a softer matrix material, shown to be critical to the cuticle's hard, yet extensible material performance (Fig. 6a)^14^,^15^. Our histological investigations provide evidence that the cuticle vesicles spontaneously coalesce into a continuous, granular surface layer on the induced core resembling a native thread (Fig. 6b,c). Notably, the cuticle vesicles begin coalescing within the ventral groove then spread over the surface of the already assembled core, indicating a clear temporal sequence of assembly. This also suggests that a specific interaction between core and cuticle vesicles might initiate assembly of the thin cuticle and that cuticle vesicle clusters may possess a low interfacial energy allowing them to spread in a fluid environment; however, presently almost nothing is known about the core-cuticle interface of native threads.

Whereas Raman spectra from cuticle vesicles and induced cuticles are rather similar, the spectrum of the native thread cuticle is strikingly dissimilar, due primarily to the presence of strong DOPA-metal coordination resonance bands (Fig. 6d). DOPA-metal interactions are critical load-bearing cross-links in the cuticle that account for more than 80% of its stiffness and hardness^16^. Thus, their notable absence in the induced thread cuticle indicates that during native thread assembly, a separate step must occur, whereby metals are infiltrated into the already assembled cuticle. Coalescence of cuticle vesicles in the absence of metal ions is contrary to the current hypothesis that DOPA-metal interactions are necessary for cuticle assembly^45^. This is a critical finding of this study, since the protein-metal co-mixing hypothesis provides the basis of current methods for producing mussel-inspired DOPA-rich metallopolymers^45^,^54^.

### Plaque formation

**Figure 7:**
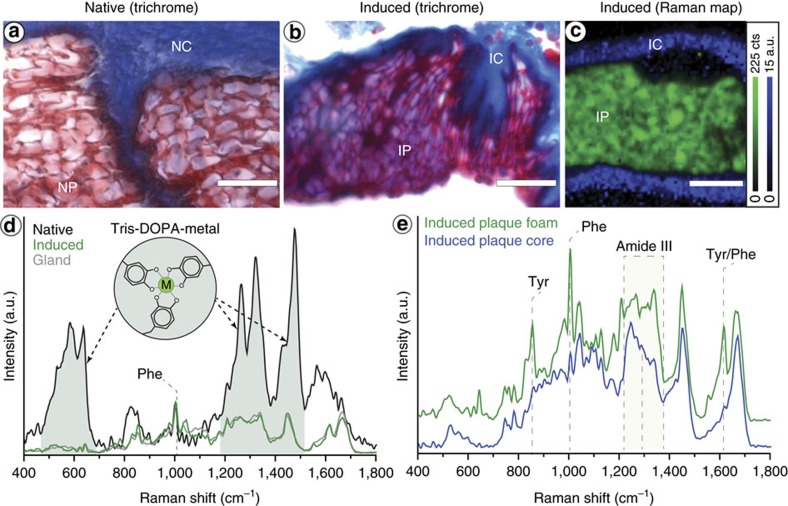
Histological and Raman characterization of induced thread plaque. (**a**) Trichrome-stained native plaque showing the interface between native plaque foam (NP) and native core fibres (NC). (**b**) Trichrome-stained induced plaque showing the interface between induced plaque foam (IP) and induced core fibres (IC). Native and induced plaques show a very similar microscale structure. (**c**) Raman confocal spectroscopic image of an induced plaque integrated over two spectral regions corresponding to the induced plaque foam (green; 1,600–1,630 cm^−1^) and the induced core fibres (Blue; ratio of integration of 1,200–1,292 to 1,292–1,377 cm^−1^). Clear separation of the core and plaque proteins is observed, confirming that precursors are stored and assemble as a dense immiscible phase. (**d**) Comparison of Raman spectra from native plaque, induced plaque and plaque gland. The intensities of the three spectra are normalized to the Phe peak height at 1,006 cm^−1^, emphasizing the much larger intensity of the DOPA resonance Raman peaks. (**e**) Raman spectra extracted from the plaque foam and core fibre regions from panel **c**. Scale bars are 10 μm for panels **a**–**c**.

The byssal thread plaque has received the most attention from researchers due to its key role in mediating underwater adhesion, which if mimicked successfully, would precipitate a number a breakthroughs in technical and biomedical applications (for example, surgical adhesives)^8^. The native plaque has a complex micro- and nano-porous architecture resembling an open-cell foam (Fig. 7a), which is believed to be a critical design feature contributing to byssus toughness and adhesive strength^17^. The plaque is comprised of at least five protein families (Table 1) that are spatially arranged through a temporal sequence of secretion^19^,^55^, with certain plaque proteins secreted directly at the surface where they initiate adhesion (mfp-3, mfp-5 and mfp-6), whereas others comprise the foamy interior (mfp-2 and mfp-4)^8^. Additionally, preCol fibres from the core are anchored into the plaque foam, creating finger-like extensions (Fig. 7a). Although previous scanning electron microscopy-based studies indicated that plaque nano-structure is not present in induced threads forcibly removed from the mussel foot^17^, we observed with histological staining of cryo-sectioned induced mussel feet that the spherical plaque vesicles spontaneously coalesce within the distal depression into a tissue with a distinctive foamy microstructure resembling the native thread plaque (Fig. 7b). Furthermore, the highly intricate, branched plaque-core interface forms spontaneously as the plaque and core vesicles interact (Fig. 7b).

Raman mapping of the plaque (Fig. 7c) reveals that the core and plaque vesicles do not mix as they coalesce – rather, they remain completely separate, with distinctive Raman spectra resembling those obtained from the core and plaque glands (Fig. 7e). The immiscibility of the core and plaque proteins is noteworthy and consistent with the hypothesis that they are stored in densely packed phases (for example, liquid crystal^32^,^33^ and coacervate^56^, respectively). Notably, the characteristic root-like morphology of the interface qualitatively resembles the phenomenon of viscous finger formation^57^, suggesting a difference in the viscosity of the phases and likely also their interfacial energies. Regardless of the mechanism, the spontaneous self-assembly of this complex interface likely plays a critical role in anchoring the core into the plaque as the vesicles coalesce and then solidify.

In addition to the morphological transformation observed in the plaque vesicles during plaque formation, a concomitant molecular level transformation is indicated by Raman spectral analysis of the intensity ratio of the two peaks of the Tyr doublet (I_850_/I_830_), which provides information about the local environment of Tyr residues present in the plaque proteins^58^. The increase in the I_850_/I_830_ ratio from 1.1 in the plaque vesicles to 1.7 in the induced plaque tissue indicates that Tyr residues transition from a primarily hydrophobic to a hydrophilic environment during thread formation^58^. This suggests that a significant conformational transformation in the plaque proteins accompanies the physical transition from a fluid vesicular phase to a solid open-cell foam, which may be relevant to the hypothesis that plaque proteins are stored *in vivo* as dense fluid phases, known as coacervates^56^. However, this possibility must be further investigated.

Comparison of induced and native plaque foam Raman spectra both reveal the presence of a strong Phe signal (1,006 cm^−1^) indicating that mfp-2 is a major component; however, the similarities end there (Fig. 7d). As with the native cuticle, the native plaque spectrum is dominated by resonance Raman bands for DOPA-metal coordination, whereas these are clearly absent in induced plaque spectra. This indicates that the vesicle-bound proteins comprising the plaque spontaneously self-assemble into a complex foamy architecture without metal coordination, contradicting existing hypotheses of byssus assembly^7^,^45^. Rather, it appears that the tendency to self-assemble is a function of the environment and structure of the proteins and that metals are added in a later finishing step (as with the cuticle), to fortify the foamy structure with intermolecular cross-links^37^. In fact, it seems likely that premature metal binding would hamper proper assembly of such complex hierarchical structures and, as recently shown with bio-inspired adhesives, would interfere with DOPA-based adhesion to surfaces^59^. Future studies will focus on better understanding how and when metal ions are introduced into the native byssus.

### Integrative model of byssus assembly

**Figure 8:**
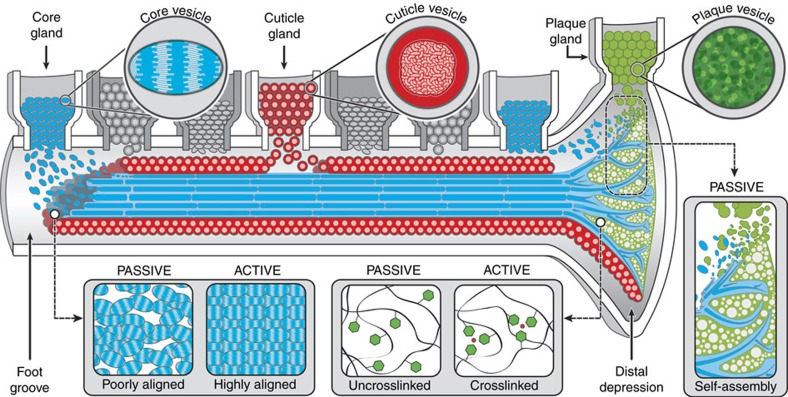
Illustrative model of passive and active aspects of byssus assembly. Byssus assembly proceeds in a manner similar to polymer injection moudling in which proteins organized in specific secretory vesicles are released into foot groove where they coalesce and spontaneously organize into native-like structures. The preCol proteins in the core vesicles are pre-organized into a liquid crystal phase, which facilitates the local organization of proteins during assembly; however, larger scale alignment of the semi-crystalline core structure likely proceeds through biologically regulated mechanical drawing. Plaque vesicle proteins are stored as a dense fluid phase that spontaneously acquires the native foam-like structure as vesicles coalesce and envelops the fibrous core, creating a root-like structure. Likewise, cuticle proteins spontaneously coalesce and spread over the core surface, creating a granular coating. Although, the cuticle and plaque require DOPA-based metal coordination for mechanical integrity, they acquire native-like structure spontaneously in the absence of metal coordination, suggesting that metal ions are likely infiltrated into the structure in a subsequent biologically regulated processing step.

The rapid fabrication of mussel byssus hierarchical structure is an extraordinary feat of sustainable bottom-up manufacturing, which offers an excellent model system for investigating biological material assembly. Our findings indicate that spatially organized vesicles in the mussel foot containing pre-packaged byssal thread precursor proteins spontaneously coalesce and self-assemble into elaborate biomolecular micro- and nano-architectures during thread biofabrication. Integrating the insights gained from our novel experimental approach with what is already known about byssal thread structure-function relationships, we propose a model of the thread assembly process, illustrated in Fig. 8.

The most important finding from the current work is the clear indication that a significant portion of the byssus assembly process is governed by basic chemical and physical phenomena–including viscosity differences, interfacial tension and liquid crystalline alignment – which can be distilled and transferred readily to the lab bench^56^. On the other hand, careful comparison of induced versus native thread structure and chemistry reveals that additional biologically regulated steps are also required for thread curing and maturation. For example, centimetre scale alignment of preCols in the core may require physical loading of fibres to align liquid crystal domains, similar to what occurs in the processing of silk^48^ or poly-aramid fibres^52^,^53^. In addition, mechanical fortification of the thread via protein-metal interactions plausibly proceeds via infiltration of metal ions following structural assembly, similar to cuticle sclerotization in certain arthropods^60^, but in stark contrast to current methods of fabricating mussel-inspired metallopolymers^45^,^54^. This multi-step curing process – reminiscent of the way a baker defines bread's internal structure by kneading and shaping the dough before baking the loaf – may be a subtle, but critical feature of the biofabrication process for achieving the remarkable structural organization and material performance of the byssus.

The interplay of physically and biologically driven processing steps in byssus formation has much to teach us about how to rapidly manufacture high-performance polymers with complex hierarchical structure under environmentally friendly conditions. These insights are especially relevant in the rapidly growing field of mussel-inspired polymers^8^,^45^,^54^, but are also broadly applicable. Once the physicochemical driving forces of assembly are better understood, for example, the fluid-to-solid transformation of byssal thread vesicles and subsequent time-dependent cross-linking in a physiological environment could inspire the development of injectable, self-assembling soft matter scaffolds (for example, for tissue repair or drug delivery^61^,^62^,^63^). Additionally, the phenomenon proposed for core-plaque interface formation, involving self-structuring of immiscible phases with different viscosities, could guide creation of complex 3D compositional and structural gradients at the interfaces of two polymeric materials. In this respect, the current study provides not only new insights into a complex and fascinating biological process, but also lays important foundation for the manufacture of next-generation polymeric material.

## Methods

### Sample preparation

Blue mussels (*M. edulis*) from the North Sea were purchased and maintained at 16 °C in an aquarium with artificial salt water. Experiments were performed in accordance with regulations of the European commission. For histological and Raman spectroscopic investigation, adult mussels of 5–8 cm length were used. Whole feet were dissected from mussels and either immediately mounted in OCT medium (VWR International) and frozen in isopentane pre-cooled with liquid nitrogen (for Raman spectroscopy) or fixed in 4% paraformaldehyde overnight before freezing (for histological staining). Using a cryo microtome (Microm HM 560) at −18 °C, 5 μm sections of frozen mussel feet were cut and then post-fixed with methanol or Buin's solution depending on the staining procedure. To investigate the dynamic thread formation process, thread production was induced by injecting a 0.56 M KCl solution in the base of the foot, as described previously in the literature^5^,^47^. Mussel feet were dissected between 5 and 10 min following induction and treated as described above to capture thread formation in progress. Histological and Raman spectroscopic investigations were each performed on eight different individual mussels with reproducible results.

### Histological staining

Histological staining on cryo-sections of native and induced mussel feet was performed using three different stains – namely, Sirius red, Masson's Trichrome and NBT. Sirius red staining, which is specific for collagen, was performed according to previous protocols^28^ in which sections were stained for 1 h in 0.1% solution of Sirius red in saturated picric acid and then washed in 0.5% acetic acid. Staining with Masson's Trichrome (Sigma Aldrich) was performed according to the manufacturer's protocol, which involved staining sections stepwise in separate solutions of Weigert's Iron Hematoxylin, Biebrich Scarlet-Acid Fuchsin, phosphomolybdic-phosphotungstic acid and aniline blue (5 min in each solution). NBT-glycinate staining, which is used traditionally to detect the presence of quinone groups (for example, oxidized DOPA residues) was performed by incubating sections in a freshly made solution of NBT (0.2 mg ml^−1^) in 2 M potassium glycinate (pH 10) for 40 min in the dark at room temperature, according to existing protocols^29^. Following staining, all sections were washed in water, dehydrated through an ascending alcohol series, cleared in Roti-Histol and mounted in Canada balsam.

### 3D model of byssus gland distribution

To construct a 3D model of gland localization in mussel feet, cryo-sections of a single mussel foot were cut every 200 μm along the length of a foot and subsequently stained with Masson's trichrome to distinguish the different glands. Using Adobe Photoshop the individual glands in the image files of each section were digitally highlighted with different grey values. The isoSurf software (version 1.5d by Graham Treece) was used to interpolate between sections generating a 3D object of the mussel foot and its glands. This 3D object was further processed with Amira software^64^ (Amira ZIB edition 2016.14, Zuse Institute Berlin). The steps of the reconstruction are illustrated in Supplementary Fig. 1.

### CT-scan

For the microCT-scan, a whole foot was dissected from a mussel, fixed in 4% paraformaldehyde overnight and stained in Lugol's solution (3.4 mg ml^−1^ iodine (I_2_) and 6.8 mg ml^−1^ potassium iodide) for 1 day. Scans were performed using a laboratory microCT (Skyscan-1072) at 80 keV, with an effective pixel size of 10 μm.

### Raman spectroscopy

Raman spectroscopy of mussel tissue and induced threads was performed on dried and unstained mussel foot cryo-sections (5 μm thickness) before and after induction with KCl. A green laser (Nd:YAG laser, *λ*=532 nm) was focused using a Confocal Raman Microscope (Alpha300, Witec, Ulm, Germany) equipped with a piezo scanner (P-500, Physik Instrumente, Karlsruhe, Germany). The scattered light was detected by a thermoelectrically cooled CCD detector (DU401A-BV, Andor, Belfast, North Ireland) placed behind the spectrometer (UHTS 300, WITec, Ulm, Germany). At least five spectra from different spots inside one gland were collected with an integration time of 60 s using a 100 × objective (Nikon, numerical aperture (NA)=0.9) and averaged. Image scans were performed at an integration time of 5 s per point. To measure the orientation of proteins in the induced thread core, image scans with polarization angles of 0° (perpendicular of the fibre axis) and 90° (parallel to the fibre axis) of the incident laser light were performed at an integration time of 3 s per point. The native core of a byssal thread was measured using hydrated 5 μm sections, and spectra from different spots were collected with an integration time of 60 s using a 60 × objective (Nikon, NA=0.8). Raman spectra of the native plaque and native cuticle were measured using hydrated 20 μm-thick thread sections; however, due to high fluorescence using the green laser, a near-infrared laser (*λ*=785 nm, Toptica Photonics AG, Graefelfing, Germany) was used in combination with a confocal Raman microscope (CRM200, WITec, Ulm, Germany) and a 60 × objective (Nikon, NA=0.8). The spectra were acquired using a CCD detector (PI-MAX, Princeton Instruments Inc., Trenton, NJ) placed behind a spectrograph (Acton, Princeton Instruments Inc., Trenton, NJ). ScanCtrlSpectroscopyPlus software (version 1.60, Witec) was used for collecting data and the OPUS software (Bruker, Germany) for spectral processing of all Raman data.

### Data availability

The data supporting the findings of this study are available from the authors upon reasonable request.

## Additional information

**How to cite this article:** Priemel, T. *et al*. Rapid self-assembly of complex biomolecular architectures during mussel byssus biofabrication. *Nat. Commun.* **8,** 14539 doi: 10.1038/ncomms14539 (2017).

Publisher's note: Springer Nature remains neutral with regard to jurisdictional claims in published maps and institutional affiliations.

## Supplementary Material

Supplementary Information Supplementary Figures

## Figures and Tables

**Table 1:** Mussel foot glands and protein contents.

**New gland names**	**Former gland names**	**Vesicle morphology**	**Potentially associated proteins**		
			**Name**	**Mass (kDa)**	**Dopa (mol %)**
Core gland	White gland	Ovoid shape	preCol-D^65^	250	<1
	Collagen gland	1–2 μm	preCol-NG^66^	250	<1
		(long axis)	preCol-P^67^	250	<1
			ptmp-1^68^	250	3
			tmp-1^47^	35	5
Cuticle gland	Enzyme glandAccessory gland	Round0.5–1 μm	mfp-1^69^	100	15
Plaque gland	Purple gland	Round	mfp-2^70^	45	5
	Phenol gland	1–2 μm	mfp-3^71^	5–7	10–20
			mfp-4^72^	80	2
			mfp-5^73^	10	30
			mfp-6^55^	11	5
