# Supplementary material for: Rapid self-assembly of complex biomolecular architectures during mussel byssus biofabrication
Source: Nat Commun. 2017 Mar 6;8:14539. doi: 10.1038/ncomms14539 (PMC5343498; doi:10.1038/ncomms14539)
Supplement: Supplementary Information — Supplementary Figures [file ncomms14539-s1.pdf]

Supplementary Information

Supplementary Figures

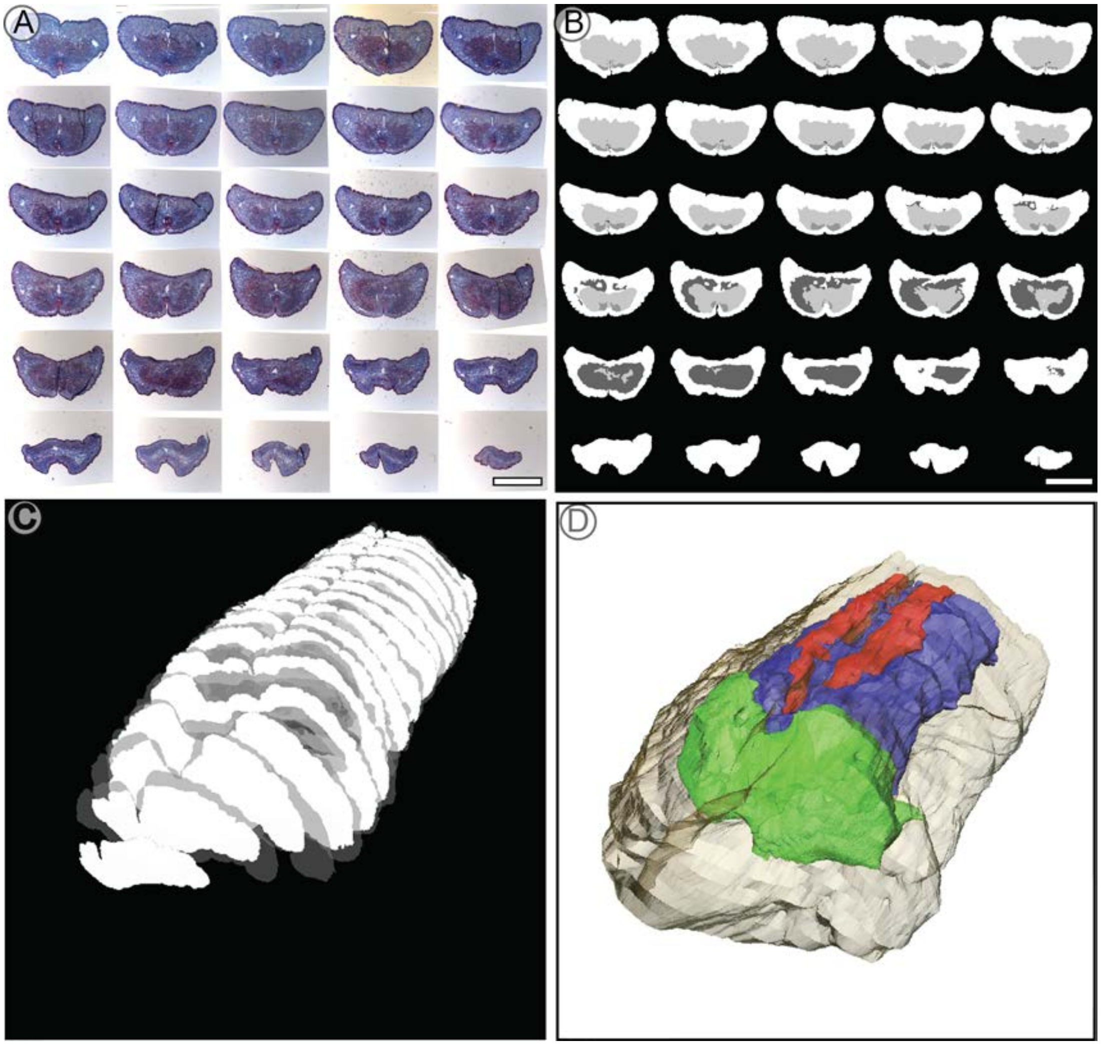

Supplementary Figure 1. Three-dimensional reconstruction of mussel foot gland localization. A) Thirty tri-chrome stained serial sections were collected every 200 μm along the length of an entire mussel foot. The top left box is the most proximal section (closest to the base of the foot) and the bottom right box is the most distal section. Sections progress from proximal to distal moving from left to right and top to bottom. B) The different glands were digitally highlighted and assigned different grey-scales. Scale bars in A and B are 2 mm. C) Processed sections

were reconstructed into a 3-D model. D) Gaps between the sections were interpolated using the isosurf software and different glands were arbitrarily assigned different colors (green = plaque gland, blue = core gland, red = cuticle gland) to produce the final 3-D model.

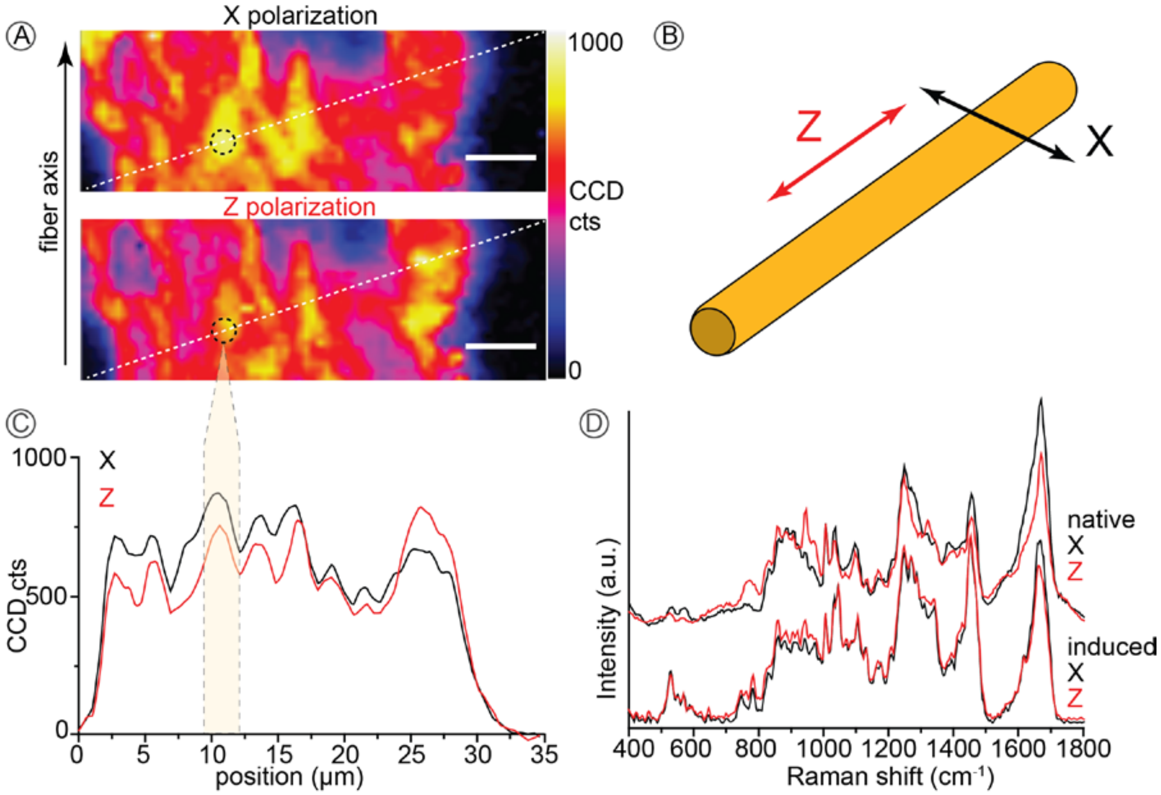

Supplementary Figure 2. Polarization dependent Raman spectra of induced threads. A) Raman images (integrated for amide I band – 1610 cm^{-1} to 1710 cm^{-1}) of a single region from an induced threads with the laser polarization oriented along the fiber axis (Z polarization) and perpendicular to the fiber axis (X polarization) as indicated in the illustration in panel (B). Local intensity variations are visible in the images, indicating differences in the integrated intensity of the amide I band. Scale bars are 5 µm. C) Plot of integrated intensity of the amide I band for X and Z

polarization along the white dashed line in panel (A), indicating that the relative intensity of the amide I band varies along the line. The observation that some regions of the induced thread show a maximum intensity of the amide I band along the Z-axis and other regions along the X-axis, indicates that proteins are not uniformly aligned along the induced thread fiber axis, in contrast to native threads. D) Comparison of polarization dependent Raman spectra from native and induced threads. Induced thread spectra was extracted from the black dashed circle in panel (A), in which preCol proteins are believed to be oriented mainly along the fiber axis according to panel (C). Induced threads exhibit smaller relative differences between the two polarizations, indicating a lower degree of protein alignment than observed in native threads.
